# Leveraging quantum computing for dynamic analyses of logical networks in systems biology

**DOI:** 10.1016/j.patter.2023.100705

**Published:** 2023-03-10

**Authors:** Felix M. Weidner, Julian D. Schwab, Sabine Wölk, Felix Rupprecht, Nensi Ikonomi, Silke D. Werle, Steve Hoffmann, Michael Kühl, Hans A. Kestler

**Affiliations:** 1 Institute of Medical Systems Biology, Ulm University, 89081 Ulm, Germany; 2 Institute of Quantum Technologies, DLR Ulm, 89081 Ulm, Germany; 3 Leibniz Institute on Aging, Fritz Lipmann Institute, 07745 Jena, Germany; 4 Institute of Biochemistry and Molecular Biology, Ulm University, 89081 Ulm, Germany; 5 International Graduate School of Molecular Medicine, Ulm University, 89081 Ulm, Germany

**Keywords:** quantum computing, Boolean networks, systems biology, quantum algorithms, cortical area development network, gene-regulatory networks

## Abstract

The dynamics of cellular mechanisms can be investigated through the analysis of networks. One of the simplest but most popular modeling strategies involves logic-based models. However, these models still face exponential growth in simulation complexity compared with a linear increase in nodes. We transfer this modeling approach to quantum computing and use the upcoming technique in the field to simulate the resulting networks. Leveraging logic modeling in quantum computing has many benefits, including complexity reduction and quantum algorithms for systems biology tasks. To showcase the applicability of our approach to systems biology tasks, we implemented a model of mammalian cortical development. Here, we applied a quantum algorithm to estimate the tendency of the model to reach particular stable conditions and further revert dynamics. Results from two actual quantum processing units and a noisy simulator are presented, and current technical challenges are discussed.

## Highlights

• Logical network dynamics were implemented on quantum processing units
• Grover’s search algorithm was used to identify predecessors of attractors
• Quantum counting was used to estimate basin sizes
• The performance of two real quantum computers was compared with that of simulators

## The bigger picture

The limits of miniaturization of semiconductor technology motivate the search for alternative hardware to further increase computational power. Quantum computing offers one such alternative paradigm. Qubits offer more degrees of freedom than classical bits, and quantum algorithms can yield speedups over classical approaches. The number of qubits on available quantum processing units has increased significantly in recent years and promises to continue to do so in the future. Systems biology, in particular the modeling of gene-regulatory networks, can benefit from these advances while retaining the simplicity of logical rules. We showcase here a proof-of-principle implementation using a biologically motivated network and highlight dynamic analyses that make use of properties such as reversibility and the existence of superposition states in quantum computing.

## Teaser

This study presents a proof of principle for implementation of the dynamics of biologically motivated logical networks using qubits. The dimension of the computational space of an entangled system of qubits matches the exponential growth of network states. Biologically relevant information about stable states is extracted using algorithms that exploit quantum properties such as superposition and are not implementable on classical hardware.

## Introduction

Many methods exist for modeling biological processes in systems biology, with model descriptions of various complexities and scales.^1^^,^^2^ These range from differential equations^3^ to Petri nets.^4^ However, Boolean networks (BNs) offer a straightforward modeling approach.^5^^,^^6^ An advantage of BNs is that they can be constructed without the need for explicitly specifying kinetic parameters, as these are often not available for all relevant interactions but still capture the main dynamics of the system.^2^ Consequently, BNs can be scaled up to include a larger number of components than other models.

In a BN with *n* components, every component has its activity represented by a time-dependent binary variable, $x_{i}\left(t\right)\in \left\{0,1\right\}$. A component may represent various entities spanning from genes, proteins, or mRNAs to entire processes such as cell death or metastasis. Each component has a regulatory function or rule of the form $f_{i}\left(x_{0},\ldots ,x_{n-1}\right)$ associated with it, which describes its regulation. These functions connect components via the Boolean operators AND (∧), OR (∨), and NOT (¬). The state of the system at time *t* is then given by a binary vector of length *n* as $x\left(t\right):=\left(x_{0}\left(t\right),\ldots ,x_{n-1}\left(t\right)\right)$.

For biologically motivated BN models, the required regulatory rules can be either constructed by extensive literature search^7^^,^^8^ or inferred directly from, e.g., gene expression data.^9^^,^^10^^,^^11^^,^^12^ To incorporate dynamics, there exist multiple update mechanisms for BNs. For instance, with synchronous updates, the rules of all *n* components are evaluated simultaneously, yielding their values at the next point in time. In contrast, asynchronous models randomly update one component at a time.

The dynamics of a BN can be represented by its state transition graph (STG).^6^ This is a directed graph of $N=2^{n}$ nodes, with every node corresponding to a state—that is, a Boolean vector of length *n*—and every edge indicating a state transition. In synchronous BNs, the system will eventually enter into a recurring cycle of stable states called an attractor, given the finite size of the STG. These can be single states (called fixed-point attractors) or multiple states (called cyclic or complex attractors). For BNs modeling biological processes, such attractors represent the system’s long-term behavior and may be interpreted as phenotypes.^13^^,^^14^ The set of states that fall into the same attractor is referred to as that attractor’s basin and can indicate the frequency of a phenotype. Attractor states of BNs have been shown to accurately capture biological phenotypes and their response to perturbations in models of various sizes and complexities.^7^^,^^8^^,^^15^

It is also possible to perturb components by fixing their state to either 0 or 1, regardless of the output of their regulatory functions. Such perturbations correspond to biological knockout (KO) or overexpression (OE) experiments.

Previous studies have extended BN models, for example, by including a continuous spectrum of activity using fuzzy logic^16^ or by the introduction of intermediate increasing and decreasing states.^17^ In a similar vein, we describe a modeling approach that aims to extend Boolean models by making use of the possibilities offered by quantum computing.

The fundamental unit of quantum computing is the qubit. In contrast to its classical counterpart, the bit, it can also assume superpositions of the orthogonal basis states, |0⟩ and |1⟩.

Consequently, a qubit’s general state |ψ⟩ is denoted by a two-dimensional state vector |ψ⟩=α|0⟩+β|1⟩ with amplitudes α,β∈C such that the state is normalized, i.e., ${\left|\alpha \right|}^{2}+{\left|\beta \right|}^{2}=1$. Once a measurement of the qubit with respect to the chosen basis is performed, its state irreversibly collapses to either of the two basis states. The probability of measuring |0⟩ or |1⟩ is given by the square amplitudes ${\left|\alpha \right|}^{2}$ and ${\left|\beta \right|}^{2}$, respectively. Thus, while the amplitudes associated with states may be complex numbers, the probabilities of measuring a particular outcome remain real-valued.

**Figure 1:**
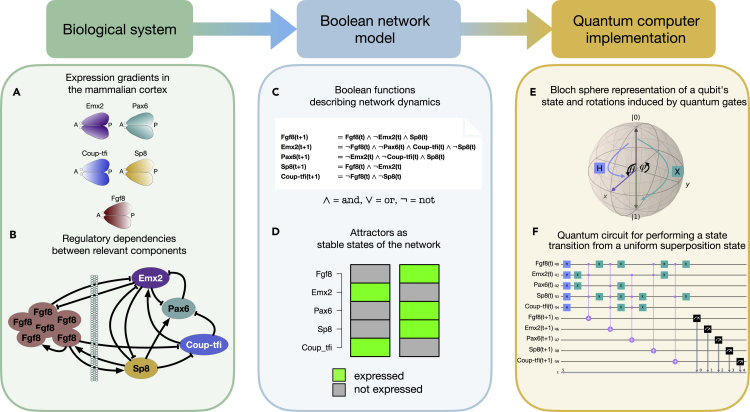
Workflow for the implementation of a quantum Boolean network (A) Expression gradients along the anterior-posterior (A-P) axis in the mammalian cortical area development network as described by Giacomantonio and Goodhill.^18^^,^^19^ (B) Activating (pointed arrows) and inhibitory (bar-headed arrows) interactions between relevant components. (C) Boolean functions specifying how to obtain the expression value of a component at the next time step in a Boolean network model. (D) Two single state attractors representing the stable states of the Boolean network. These states correspond to biological phenotypes. (E) States of a single qubit can be represented on the Bloch sphere. In addition to the classical binary states, this allows for the creation of superposition states. Quantum gates such as the X or H gate correspond to rotations of the qubit’s state on the Bloch sphere. (F) Creation of a quantum circuit from a series of quantum gates. This circuit performs a state transition on a uniform superposition of all $2^{n}$ states of the same network and measures its output.

Choosing the parameters α=cos(θ/2) and $\beta =e^{i\varphi }\;\sin\left(\theta /2\right)$, the state |ψ⟩ can be expressed in spherical coordinates as |ψ⟩=|ψ(θ,φ)⟩ with θ∈[0,π] and φ∈[0,2π). A common visualization of a qubit’s state is shown in Figure 1E, where the state is represented by a vector on the surface of a Bloch sphere. When identifying the classical states 0 and 1 with the basis states |0⟩ and |1⟩, the corresponding points on the Bloch sphere are the poles on the z axis, whereas the points on the equator are equally weighted superpositions of the basis states. The angle θ can be tuned to yield a superposition of the basis states with arbitrarily chosen weights.

The same principles apply to entangled multi-qubit systems. For n=2 qubits, for example, quantum states can be superpositions of up to four basis states, i.e., |ψ⟩=α|00⟩+β|01⟩+γ|10⟩+δ|11⟩ with α,β,γ,δ∈C and ${\left|\alpha \right|}^{2}+{\left|\beta \right|}^{2}+{\left|\gamma \right|}^{2}+{\left|\delta \right|}^{2}=1$. In this manner, the dimension of the computational space in which the quantum state lives, called the Hilbert space, grows exponentially. The basis states of a superposition can then be identified with the corresponding bitstrings denoting the expression of genes or lack thereof at some time point *t* in a BN.

The required Boolean logic can be implemented in quantum circuits, i.e., sequences of gates applied to a register of qubits. Gates can be applied to single qubits, such as the Hadamard gate, or multi-qubit gates, like a controlled-NOT (CX), which create entanglements between qubits. Relevant gates are listed in section 1 of the supplemental information. All presented quantum circuits are reversible. This property is equivalent to their unitarity, a necessary constraint on gate operations.^20^

One of the most prominent algorithms in quantum computing is Grover’s search algorithm.^21^ The goal of this algorithm is to search for a marked item ω (or set of *M* items) among an unsorted set of *N* elements. By exploiting quantum properties through a procedure called amplitude amplification,^22^ Grover’s algorithm has a complexity of $O\left(\sqrt{N/M}\right)$,^20^ providing a quadratic improvement over a classical search. In the course of our investigation we will also utilize this procedure.

Quantum computing may mark a new era of computation power. We aim to investigate the opportunities for systems biology approaches using this paradigm. While quantum computing has already found applications in other areas of biology,^23^^,^^24^^,^^25^^,^^26^^,^^27^ we aim to evaluate the possibilities for using quantum computing for BNs as dynamic models of biological systems. Recent work by Qi et al. has demonstrated how Boolean time series can be generated by periodic quantum measurements resulting from a continuous Lindblad master equation based on an interaction graph.^28^^,^^29^ In contrast, we focus on the analysis of biological networks with known and fixed update rules, tailoring quantum algorithms to extend the analysis of STGs.

Thus, our work aims to transfer the classical modeling approach to quantum BNs (QBNs), showcasing how quantum hardware and algorithms are suitable for solving problems in the analysis of BN dynamics, while retaining the simplicity of Boolean logic.

In this work, our analysis will focus on an n=5-component BN modeling mammalian cortical area development, as presented by Giacomantonio and Goodhill.^18^^,^^19^ The comparison of this analysis to the known outcomes from classical BNs aims to be a proof of principle that quantum computing can be applied to problems of this kind. Figure 1 highlights the components included in this model as well as the role of their expression gradients in the specification of the anterior-posterior axis of the mammalian cortex. The network is represented by a set of Boolean functions, which are then translated into a quantum circuit.

## Results

The extension toward QBNs requires the implementation and analysis of quantum circuits generated from a set of classical Boolean regulatory functions.

While Franco et al.^30^ have investigated random BNs using quantum Boolean functions with asynchronous updating, our work focuses on the relevance of quantum computing for the analysis and interpretation of biological networks and their attractors. As a proof of principle, we demonstrate dynamic analyses on small models, which show a scale-free topology typical of gene-regulatory networks.^31^ Our analysis is specifically focused on attractor states, their surrounding states in the STG, and their basin sizes. While there are algorithms to screen for attractors in larger BNs,^17^^,^^32^^,^^33^ the exponential growth of the number of nodes in the STG prohibits its complete exploration as well as the identification of basins of attraction. This is a constraint for different kinds of analyses in biomedical research, such as screening for therapeutic targets and their impact. Since the dimension of the Hilbert space of a multi-qubit system likewise grows exponentially, the $2^{n}$ possible states of a BN can be encoded in the basis states of *n* qubits. Consequently, the exponential growth of complexity is reduced to a linearly growing demand for the number of qubits. First, we demonstrate how a uniform superposition state converges to a superposition of attractor states. This is followed by simulations showing how convergence differs when making use of parameterized rotation gates to continuously tune the initial activity of network components. We further show how perturbations with superposition states affect dynamics.

In addition, two existing quantum algorithms are applied to biologically motivated networks. Specifically, Grover’s search algorithm^21^ is adapted to identify predecessors of a marked state up to any arbitrary number of previous time points. Similarly, a quantum counting algorithm^34^ is used to directly estimate the total number of these predecessors.

Last, we run experiments on two real quantum processing units, one based on trapped ions and one based on superconducting qubits, to compare the impacts of differences in noise, transpilation, and qubit connectivity on a quantum state transition.

### Generation of circuits for multiple state transitions

As a first step, a text file specifying the regulatory rules of a network as it is used in the R package BoolNet^35^ is parsed into a quantum circuit performing a single state transition, as shown in Figures 1C and 1F. For a network of *n* components, this circuit will have 2n qubits. The first *n* qubits serve as inputs on which the quantum gates act. The second set of *n* qubits yields the measurable output of the system. Notably, the circuit structure also leads to an inherent parallelization in the evaluation of the Boolean functions.

In a preliminary step, a layer of Hadamard gates is composed onto the first *n* input qubits. This initialization serves to create a uniform superposition state ${|\Psi_{H}\rangle }_{n}$ of all $N=2^{n}$ basis states:

(Equation 1) $${|\Psi_{H}\rangle }_{n}=H^{⊗n}{|0\rangle }_{n}=\frac{1}{\sqrt{2^{n}}}\sum_{i=0}^{2^{n}-1}{|\psi_{i}\rangle }_{n}\text{,}$$

with ${|\psi_{i}\rangle }_{n}$ indicating the *n*-qubit state whose bitstring representation corresponds to the integer *i*.

That is, the system starts in a superposition state of maximal uncertainty and performs *N* classical state transitions simultaneously, with any measurement collapsing the wave function to yield one particular successor state. The probabilities for a given output are determined by the structure of the network’s STG.

For the sake of simplicity, in the following, all state transitions are assumed to be synchronous. However, it is also possible to conduct asynchronous transitions by shifting the order of applied gates and considering whether their inputs should come from the first or second register of qubits in a transition circuit. An example of this is shown in section 2 of the supplemental information.

The implemented scheme for conducting *T* repeated state transitions uses a single large circuit of (T+1)n qubits, with each transition having a separate register of qubits. See also section 3 of the supplemental information. The average transient time to attractors in scale-free networks such as biological systems increases linearly.^31^^,^^36^^,^^37^ This increase results in a demand for qubits scaling as $O\left(n^{2}\right)$ if one desires to capture trajectories along the entire transient time.

For a single transition, the shifts in the probabilities of states can be summarized as follows. Assuming that every state *i* has some probability weight $w_{i}^{t}$ associated with it at time *t*, the transition operation $\hat{T}$ shifts and sums up these weights in the output of each node in the STG. That is, the probabilities for measuring a given state in the output register t+1 will change from having only the default bitstring of zeros (i.e., $|\psi_{0}\rangle =|000\ldots 0\rangle$) as a possible outcome, as described by Equation 2:

(Equation 2) $${|\psi_{0}\rangle }_{t+1}\langle \psi_{0}|\to \sum_{i,j\in S}\delta_{\text{succ}\left(j\right),i}{\left|w_{j}^{t}\right|}^{2}{|\psi_{i}\rangle }_{t+1}\langle \psi_{i}|\text{.}$$

Here, S denotes the set of all states, $\delta_{\text{succ}\left(j\right),i}$ is the Kronecker delta, and succ(j) is the integer representation of the successor state of the bitstring corresponding to the integer *j*. For example, in a 3-qubit system, the basis state $|\psi_{7}\rangle$ would correspond to the bitstring 111.

### Convergence of a uniform superposition state to attractors

Attractor screening is an essential part of the analysis of BNs. We perform state transitions on superposition states to enable a quantum-specific search of attractors. These circuits will also be used as building blocks for the implementation of quantum algorithms. Assuming the absence of noise, and since all nodes in the STG have exactly one successor state in a synchronous update scheme, this weight shift is deterministic. Thus, after the first transition, all Garden of Eden (GoE) states,^38^ i.e., states that have no incoming edges in the STG, will no longer be possible results of a measurement of the final *n*-qubit register. In this manner, every transition reduces the number of possible outcomes until only attractor states remain. To evaluate our QBN-based attractor search, we applied it to the described example of the mammalian cortical area development network.^18^ The results of this simulation were then compared with the known attractors using the classical approach. In a classical simulation, this network possesses two single state attractors, 10010 and 01101, partitioning the STG into two basins making up 87.5% and 12.5% of the nodes, respectively.

**Figure 2:**
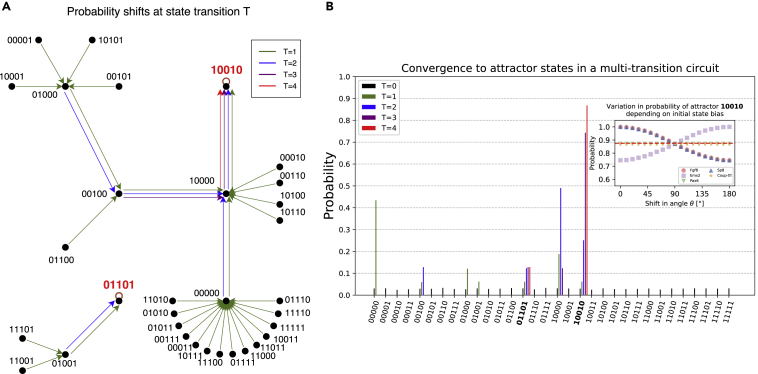
Dynamic simulation of a quantum Boolean network (A) State transition graph of the mammalian cortical area development network.^18^ The color coding indicates weight shifts that occur simultaneously in the *T*-th transition of a quantum circuit, starting from a uniform superposition state. (B) After T=4 transitions, only the two single state attractors remain as possible outcomes of a measurement with their probabilities corresponding to their basin sizes. The use of tunable $R_{y}\left(\theta \right)$ gates instead of H gates in the initialization may change these probabilities for some components. The dashed red line in the inset indicates the basin size of the 10010 attractor in the classical Boolean network. For every component, biased $R_{y}\left(\theta \right)$ gates were used for initialization while keeping an unbiased H-gate initialization for all other components. This reveals the components whose activity has an impact on the basin size. See also Figure S4.

Figure 2A shows the complete 32-node STG of this network given synchronous updates, with arrows of a given color indicating simultaneous probability shifts occurring at a quantum state transition. A barplot visualizes the increase and decrease in the probabilities for measuring any state after some fixed number of transitions *T* starting from a uniform state ${|\Psi_{H}\rangle }_{n}$. To account for the stochastic nature of measurement, a large number of 10,000 measurements have been simulated for each value of *T*. After T=4 transitions, only the two attractors remain. These were obtained with probabilities of 87.0±0.3% and 13.0±0.3%, which nearly match the classical simulation. Errors were calculated as specified in Equation 6 in the experimental procedures.

### Identification of components determining long-term behavior

In classical BNs, components affecting the resulting set of attractors need to be searched by fixation of each component to 0 and 1. Quantum hardware is a natural fit for this kind of analysis since it allows one to determine this information without additional simulation effort. To do so, we adapted the circuits used in the previous section. Instead of initialization with a layer of Hadamard gates, it is also possible to use parameterized $R_{y}\left(\theta \right)$ gates, thus biasing the expression of genes in the initial state. This yields the same attractor states as in the unbiased network; however, the probabilities of these states will be shifted. It is therefore possible to bias components toward being inactive (θ∈[0,π/2)) or active (θ∈(π/2,π]) on a continuous spectrum. A choice of θ=π/2 will yield the same results as the use of a Hadamard gate, giving the unbiased basin sizes as found in a classical BN. In contrast, the edge cases of θ∈{0,π} will yield the basin sizes obtained when sampling only those states of the STG where the given component is off (or on).

The inset of Figure 2B shows how the attractor distribution is affected by biasing single components in the network. Ten thousand measurements were simulated for every component and every value of θ. Notably, only some components affect the distribution, while it remains unchanged for others, regardless of bias.

A similar analysis was performed for the cell-cycle network of Fauré et al.,^15^ containing *n* = 10 components, using a different state transition circuit. Results are shown in section 4 of the supplemental information.

### Effect of perturbations using superposition states

A typical setting for BNs is the evaluation of perturbation experiments such as knockout mutations. For QBNs, we developed an approach to perform perturbations using superposition states. Instead of updating perturbed components, the qubits carrying their state at the initial time *t* are reused at every transition. This can be done for multiple components at the same time. For *P* components to be perturbed, the resulting attractor distribution of this simulation resulted in the union set of attractor states from all $2^{P}$ combinations of overexpressions and knockouts of these *P* components. As an example, in our model of cortical area development,^18^ we perturbed P=2 components: Pax6 was perturbed with a bias toward overexpression using an $R_{y}\left(\theta =3\pi /4\right)$ gate in the initialization of the circuit, while Coup-tfi was biased toward knockout using an $R_{y}\left(\theta =\pi /4\right)$ gate.

These two components were chosen because every one of the four possible double perturbations leads to at least one attractor that is unique to that particular perturbation and does not occur in the other three. Section 5 of the supplemental information lists the resulting attractors and their probabilities.

Since it is possible for a specific attractor to appear as the result of multiple perturbations, this method can be used to more directly screen the total set of attractors resulting from $2^{P}$ classical simulations.

### Identifying predecessors of marked states using Grover’s algorithm

Quantum circuits not only allow one to re-create experiments with the classical BN setting. On top of that, the QBN model provides access to a separate class of quantum algorithms. Since attractors correspond to phenotypes, it is biologically interesting to investigate sets of predecessor states that may be initial conditions leading to a given attractor. This can be achieved using Grover’s algorithm, exploring the STG from the attractor outward, and thus giving a special focus to the states closest to the system’s phenotype. The number of transitions performed in this inverted direction will be denoted as $T^{inv}$.

Grover’s algorithm^21^ is a quantum algorithm that searches for a marked element in an unstructured database of size *N*, offering a quadratic improvement in complexity relative to classical approaches. The general procedure behind this algorithm is known as amplitude amplification.^22^

We apply this algorithm to BNs, using an attractor or other state of interest as the marked element. A uniform superposition state is used as the initial search state, in which the amplitude of the solution states will be amplified.

The black box oracle operation in Grover’s algorithm is implemented via Boolean state transitions. The phase difference resulting from the marking of an attractor is carried back to previous registers via uncomputing. Thus, predecessor states can be amplified. The circuit for performing this task is shown in section 6 of the supplemental information.

For all searches, 10,000 measurements were simulated for Grover circuits based on transitions in the network of Giacomantonio and Goodhill.^18^

There is an optimal number $G^{\text{opt}}$ of iterations of the Grover operator *G* to perform, which leads to a high amplitude of the solution state.^39^ This number depends on the number of solutions *M*, that is, the number of marked elements, and the size of the state space *N* as:

(Equation 3) $$G^{\text{opt}}=\left\lfloor \frac{\pi }{4}\sqrt{\frac{N}{M}}\right\rfloor \text{.}$$

In an exemplary search for the immediate predecessors of the attractor 01101 with G=1 iteration of the Grover operator, a total of 47.41%±0.5% of measurements yielded either the attractor itself or its predecessor 01001 (96.12%±0.2% using $G_{T^{inv}=1,01101}^{\text{opt}}=3$), even though these states make up only 6.25% of the state space. When searching for all $T^{inv}=2$ pre-predecessor states, the four states in the basin of the 01101 attractor (12.5% of the total state space) were obtained with a cumulative probability of 77.68%±0.4% (94.60%±0.2% when using $G_{T^{inv}=2,01101}^{\text{opt}}=2$). Since the marked attractor is a single state attractor, all solutions are equally amplified in the case of multiple solutions. In the general case of cyclic attractors, amplification may depend on which state in the cycle was marked.

In particular, when the marked state is a GoE state, there is no solution that yields this state as a successor. Consequently, there is no amplitude amplification taking place, and the circuit will return the same uniform superposition that was given as an input.

Since the presence of Boolean functions imposes structure on this search problem, the actual complexity of the search will depend on the specific network in question. For example, if the network includes inputs, meaning components that are themselves unregulated or regulated only by themselves, then the state of these components necessarily remains fixed once set. To account for this, the initial search state can be set to any arbitrary distribution, e.g., having input components set to |0⟩ or |1⟩ instead of using a superposition via a Hadamard gate.^40^ This will change the structure of the search space and can be used to include prior available knowledge about predecessor states.

If the number of solutions *M* is unknown, a quadratic speedup can still be achieved by applying a generalized version of Grover’s algorithm, which adaptively increases the number of Grover iterations.^39^

### Estimating the number of predecessor states using quantum counting

Quantum counting^34^ is an algorithm that relies on amplitude amplification to estimate the number of solutions *M*.

**Figure 3:**
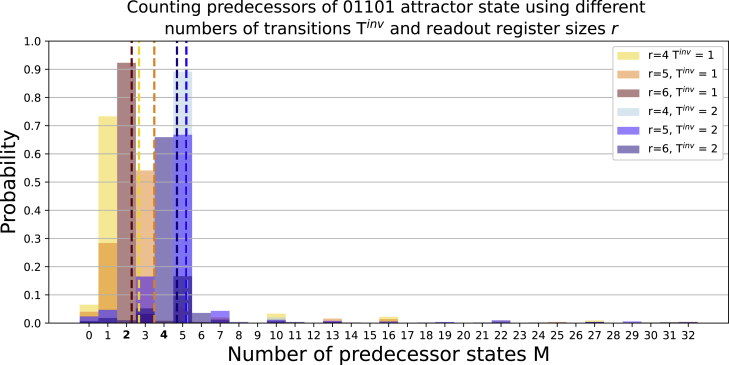
Use of quantum counting circuit to establish the number *M* of predecessor states of a desired state of interest at some number of inverted transitions $T^{inv}$ A quantum counting circuit has been run to establish the number of predecessor ($T^{inv}=1$) and pre-predecessor ($T^{inv}=2$) states of the attractor 01101 in the mammalian cortical area development network.^18^ This yielded results close to the values of M=2 and M=4, respectively, which are expected from a classical simulation of the STG. The dashed vertical lines indicate the means of the corresponding probability distributions. In general, the accuracy of these results may be improved using more iterations of the Grover operator and their associated readout qubits *r*.

It uses a version of the Grover operator in combination with an additional register of *r* control qubits serving as readouts. Any measured outcome of this register corresponds to a particular integer value for the estimation of *M*. Figure 3 shows the results of a quantum counting circuit being measured 1,000 times each for different sizes *r* of the readout register. The circuit itself as well as the calculation of *M* from the measured outcomes is shown in section 7 of the supplemental information. Referring to the STG of the cortical area development network shown in Figure 2A, this allowed for the identification of the basin size of M/N=4/32 for the 01101 single state attractor by using a circuit to perform $T^{inv}=2$ inverted state transitions as part of the Grover operator. Likewise, the two immediate $T^{inv}=1$ predecessors of this attractor could be identified. Since the marked state was a single state attractor, a counting circuit for $T^{inv}$ steps will also include all solutions for $\left\{1,\ldots ,T^{inv}-1\right\}$ steps.

To quantify the growth in the number of predecessors in biological networks, we investigated a set of 28 published networks (these are listed in section 8 of the supplemental information). For each network, the full transition table for all $2^{n}$ states was generated to calculate the ratio M/N starting from any attractor state. For a single inverted transition $T^{inv}=1$, a median value of M/N=0.003 (IQR = 0.023) was found across all attractors of all networks. Moreover, there was a Pearson correlation of −0.714 between the average M/N for immediate predecessors in a given network and network size. This indicates that larger networks may fall into the regime of small M/N values in which the advantages of quantum search algorithms are most notable over classical approaches.

### Impact of noise on a simulator and a real quantum processing unit

Real quantum computers are exposed to various kinds of noise. These include decoherence and thermal relaxation as well as gate and measurement errors.

Furthermore, to adapt to a specific processor, a quantum circuit has to be transpiled. That is, the sequence of quantum operations defined by the algorithm needs to be translated into a sequence of quantum gates that are natively available on the hardware. In particular, it is often not possible to implement 2-qubit gates on any arbitrary pair of qubits. The set of all possible connections in a system is specified by an undirected graph, called its coupling map. To apply 2-qubit gates on a pair of qubits that are not directly connected, it is necessary to swap connected qubits until the two target qubits are connected. This increases the circuit’s depth.

To evaluate whether current quantum computers are suitable substitutions for classical BN analysis, we measured how the setup is influenced by noise and error rates in current state-of-the-art quantum processing units. We performed a single state transition starting from a uniform superposition as shown in the circuit of Figure 1F on a noiseless as well as a noisy simulator. For this, we chose the “FakeToronto” mock backend offered by Qiskit. This backend mimics the constraints of the real 27-qubit Falcon r4 IBMQ Toronto processor.

The coupling map of this processor contains a total of 28 connections, leading to a connectivity of $\frac{28}{\left(1/2\right)\cdot 27\cdot 26}$ = 7.98%.

Experiments were also run on the real IBMQ Toronto processor, adding schemes for dynamical decoupling^41^ as well as readout error mitigation.^42^ In addition, we transpiled the same transition circuit to fit the set of gates available on the 11-qubit trapped ion processor of IonQ^43^ and ran experiments on this device using Amazon Braket’s cloud service. This system has a completely connected coupling map.

The maximal number of gates that must be executed in sequence inside a circuit is the circuit’s depth. Transpilation to the IBMQ Toronto backend resulted in a circuit with a depth of 261. In contrast, transpilation for the IonQ processor yielded a circuit depth of 140.

**Figure 4:**
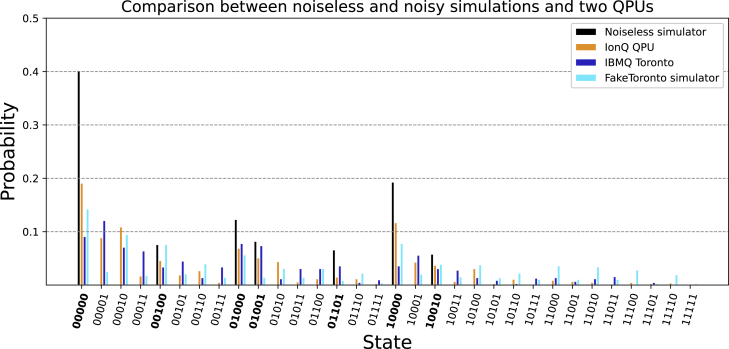
Comparison of measurement distributions of a noiseless simulator with a noisy mock backend and two real quantum processing units Barplot showing the probabilities of measuring any given state after a single quantum state transition starting from a uniform superposition in the mammalian cortical area development network. Shown are a noiseless simulator, a noisy simulator emulating properties of the IBMQ Toronto processor, a trapped ion-based processor from IonQ, and the real superconductor-based IBMQ Toronto processor.

Figure 4 shows the distributions of measured states for these experiments.

A classical simulation shows that after this single state transition, 7 of 32 states should remain as possible measurements, the most probable one being the state 00000 with a weight of 43.75%.

In all four cases, a total of 1,000 measurements were performed.

The similarity of the probability distributions in Figure 4 was quantified using a normalized fidelity measure $F_{\text{normed}}\left(P_{\text{ideal}},P_{\text{output}}\right)$ between an ideal probability distribution and the actual obtained output^44^ as described in Equation 5 in the experimental procedures.

Taking the noiseless simulator to yield the ideal distribution, the resulting fidelities are listed in Table 1.

**Table 1:** Normalized Hellinger fidelities

IonQ	IBMQ Toronto (mitigated)	IBMQ Toronto (unmitigated)	FakeToronto
0.405	0.196	0.114	0.250

Results comparing a noiseless simulator (ideal) with a noisy simulator as well as with two real quantum processing units (output) for a single transition in the cortical area development network.^18^

Notably, the real IBMQ Toronto processor achieved a worse fidelity with respect to the distribution of the noiseless simulator than its mock backend equivalent. A potential cause for this was pointed out in a recent benchmarking study by Lubinski et al.^44^ The authors state that due to the various and complex sources of errors, qubit-specific metrics (i.e., measures such as decoherence times or gate error rates as used by the noise model of the mock backend) are not sufficiently predictive for performance. Another potential cause of this drop in performance relative to the mock backend may be the time that has passed since the last calibration of the device.

## Discussion

In this study, we have explored possible ways to extend the simulation of biological BNs to quantum computers. Molecular biology may benefit from quantum BNs for the following reasons.

First, the growth of dynamic state spaces is matched by the growth of the corresponding quantum systems’ Hilbert space. The exponential speed at which the state spaces of BNs grow poses a challenge to their exhaustive simulation using classical processors. However, the dimension of the Hilbert space of a multi-qubit system likewise grows exponentially. Thus, quantum processors may be a more suitable hardware for exploring high-dimensional spaces requiring a linear increase in the number of qubits, although in general it may be necessary to perform an exponential number of measurements to achieve the desired information.

Second, we have an extension of the classical binary by introducing superposition states that can be both 0 and 1. While the resulting outcomes themselves remain binary once measured, their amplitudes in the superposition can take on continuous values. The modeler may tune these to modify the expression of nodes of interest based on prior knowledge or for hypothesis testing. This tuning results in an alteration of the probabilities of the attractor landscape. Thus, we can retain the simplicity of Boolean logic without integrating kinetic parameters. For example, components representing processes like cell death, e.g., in a cancer network, may be biased to 0 in an initial state. This does not perturb the network, but leads to the exploration of a particular subspace, where any cell death is a consequence of dynamic state transitions. Thus, specific interactions can effectively be assigned more or less importance in a manner that is naturally implemented on quantum hardware. The biasing of initial states may further be applied in the case of networks that had their rules inferred from expression data.^9^^,^^10^^,^^12^ In this case, the expression counts could be rescaled toward the (0,1) interval and used to obtain a more realistic weighting of basins.

Third, perturbations with superposition states of *P* components can return the union set of attractors resulting from $2^{P}$ classical simulations, allowing for a simpler and more direct screening of phenotypes due to the potential overlap of resulting attractors between perturbations.

Furthermore, we might exploit a complexity advantage due to quantum algorithms. By reformulating the dynamics of a BN as a search problem, one can use quantum subroutines such as the amplitude amplification in Grover’s algorithm. This may provide advantages relative to classical approaches, especially when the ratio of solutions to the size of the state space M/N is small. We have shown indications that this ratio may decrease in the search for immediate predecessors of an attractor as network size increases. Moreover, recent research has yielded alternative schemes for the quantum algorithms used,^45^^,^^46^ which may further decrease the depth of the resulting circuits.

Last, there is the possibility of the inversion of dynamics. The combination of superposition states and the reversibility of quantum circuits allows the implementation of quantum algorithms that can backtrace the STG. This is challenging to analyze on classical hardware since it does not inherently possess the reversibility property that is characteristic of quantum circuits. Biologically, this can identify the number and kind of initial states that lead to a given outcome. For example, suppose the final attractor shows a pathological activation pattern of some component. In that case, one can use these inverted transitions to trace back to where this activation first occurred and to ultimately reconstruct related molecular mechanisms. Attractor states correspond to the long-term behavior of a biological system, while states far away from them are more transient. Thus, the inversion of the direction of dynamics made possible by quantum algorithms can be applied to more efficiently explore the immediate surroundings of an attractor and search for patterns in these states. It might also be possible to use a similar circuit to identify predecessors of any state where a particular component is on or off, which would not require previous knowledge of attractors.

Regarding the choice of hardware, many different implementations for quantum processors are currently being investigated. A recent benchmarking study by Lubinski et al.^44^ gives an overview comparing various state-of-the-art processors.

While there is no hardware that can be considered clearly superior among these technologies, certain approaches have shown advantages over others in specific areas. For example, it has been demonstrated that trapped ions have the potential to achieve coherence times much longer than those in competing systems.^47^ Furthermore, in principle, any component in a BN can be regulated by any other. Therefore, a complete coupling map is a highly desirable property for the implementation of QBNs. Moreover, an upcoming next-generation system by IonQ claims an order of magnitude reduction in gate errors relative to the one used in this work.^48^

Finally, on future quantum hardware one may be able to use quantum error correction, which allows one to reduce or even fully remove the effects of noise on the qubits by encoding a logical qubit in multiple physical qubits.^49^

It may be possible to further optimize the construction of the state transition circuits for the case of more extensive networks, which may include more complex Boolean functions with a larger number of regulating components. Here, it may be helpful to add ancillary helper qubits to store the results of subfunctions that occur across multiple rules, leading to broader but shorter circuits. For a general procedure for robust quantum computing, Rieffel and Polak^50^ outlined how a circuit can be made more robust even at the cost of increased size.

One may further improve the algorithms used in this work for future research. For example, modified algorithms such as fixed-point amplitude amplification^51^^,^^52^ allow for target states to be reached exactly, reducing the number of measurements required to obtain the entire solution set. Furthermore, it is to be noted that amplitude amplification is a subroutine that can yield quantum speedup for problems other than unstructured search. One particular problem where this is the case is Boolean satisfiability (SAT), as a quantum algorithm could further improve the complexity of Schöning’s algorithm.^53^^,^^54^ While classical heuristics are currently still more scalable than quantum search, it is thus possible that these heuristics may themselves benefit from a quantum implementation. Regarding BNs, this is especially interesting concerning the SAT-based attractor search algorithm of Dubrova and Teslenko^32^ used in BoolNet.^35^

Other methods generating Boolean dynamics based on quantum measurements such as those of Qi et al.^28^^,^^29^ may also be applicable in a biological context. For example, one may run a reconstruction algorithm on the time series resulting from the jumps between states along a Markov chain^12^ to derive sets of Boolean functions fitting these dynamics. Alternatively, interaction graphs from databases such as STRING-DB^55^ might be given as input to obtain the matrix of probabilities describing the Markov chain. Then, a second run on a modified graph in which key edges have been removed or putative interactions added yields a second matrix. One could then compare these matrices regarding the probabilities for states to jump into a desired attractor or subspace.

To conclude, we have shown that the analysis of biologically motivated BNs could be a suitable application for the growing possibilities offered by quantum computing. The proposed QBN approach is able to capture the behavior of classical BNs with synchronous or asynchronous update schemes while offering further possibilities through the use of quantum algorithms.

While Moore’s law is reaching its end due to the limits of miniaturization,^56^ the number of qubits in IBM’s quantum processing units (QPUs) has increased exponentially in recent years. Starting with 5 qubits in 2016, currently available systems have reached 127 qubits. So far, every milestone in the IBM development roadmap has been achieved, and future releases are planned for devices including up to 4,158 qubits in 2025.

Given existing processors, the immediate predecessors of attractors may be analyzed for networks up to n=63, requiring 2n+1 qubits. The entire basin may be amplified up to n=11 assuming a transient time equal to *n*.^36^

However, such circuits will require active error correction to yield useful results. A well-known approach able to correct single-qubit errors is Shor’s 9-qubit encoding.^57^ This would reduce the possible analyses down to n=7 for immediate predecessors and n=3 for full basins.

On a future 4,158-qubit processor, the same circuits could be implemented for n=2,078 and n=230 in uncorrected circuits and n=64 and n=21 for a 9-qubit encoding. Again, this highlights the trade-off between using additional qubits to analyze longer trajectories and implementing necessary error correction. In addition to more sophisticated error mitigation and active correction, a reduction of multi-qubit cross talk errors will be required to analyze networks of these sizes given the large number of entangled qubits.

## Experimental procedures

### Resource availability

#### Lead contact

The lead contact for this work is Hans A. Kestler (hans.kestler@uni-ulm.de).

#### Materials availability

There are no physical materials associated with this study.

### Method details

Simulations were performed using Qiskit v.0.36.2^58^ and Python v.3.9. All states are denoted in the 0-indexed little-endian format used by Qiskit. For example, the 3-qubit state given by $|q_{0}\rangle =|1\rangle ,|q_{1}\rangle =|0\rangle ,|q_{2}\rangle =|0\rangle$ is written as |001⟩.

#### Generation of circuits

Boolean functions are parsed into circuits using the ClassicalFunction compiler available in Qiskit. The Boolean rule of each component is synthesized into a separate circuit with *n* inputs and 1 output. These circuits are then composed to yield a circuit that updates all components either synchronously or asynchronously.

#### Network selection

The networks used for the calculation of the ratio M/N after a given number of inverted state transitions were extracted from https://cellcollective.org/^59^ as well as from PubMed by using the search term “Boolean network model” (status 24.05.2017).

For the calculation of the reduction speed in possible measurement states after some number of transitions, we analyzed the transition tables of the networks using the BoolNet R-package.^35^ Since exhaustive attractor searches are limited to at most n=29 components in this package, only networks that did not exceed this limit were analyzed.

We further excluded networks for which analyses of the full transition table could not be conducted in under 24 h of computation time.

Networks were also not considered if their dynamics could be reduced to a set of input components. Last, the PoweRlaw R-package^60^ was used to check for scale-free degree distributions, retaining networks with p values above a threshold of p = 0.1 as described by Clauset et al.^61^

In total, this set of networks has an average of 15.5±5.4 components, with an average of 38.6±17.1 interactions.

Section 8 of the supplemental information lists further details regarding these networks.

#### Quantification of noise

Repeated measurements of quantum circuits will yield discrete probability distributions over all $2^{n}$ possible states of a QBN. The similarity between two such distributions, *I* and *O*, e.g., one from an ideal noiseless simulator and one as output from a noisy quantum processing unit, is quantified using the fidelity $F_{s}\left(I,O\right)\text{:}$

(Equation 4) $$F_{s}\left(I,O\right)={\left(\sum_{s\in S}\sqrt{p_{I}\left(s\right)p_{O}\left(s\right)}\right)}^{2}\text{,}$$

as given in the benchmarking study of Lubinski et al.,^44^ with S denoting the set of all possible states.

This measure is then normalized to $F_{\text{normed}}\left(I,O\right)$ as defined by Lubinski et al.^44^ so that comparisons to a uniform probability distribution *U* over the set of states S will be mapped to a value of 0:

(Equation 5) $$F_{\text{normed}}\left(I,O\right)=\frac{F_{s}\left(I,O\right)-F_{s}\left(I,U\right)}{1-F_{s}\left(I,U\right)}\text{.}$$

For simulations in which a number of measurements *m* have been performed to obtain a probability *p* of a given outcome such as an attractor state, the error is calculated as:

(Equation 6) $$\varepsilon =\sqrt{\frac{p\cdot \left(1-p\right)}{m}}\text{.}$$

## Data Availability

The code for performing the analyses shown in this work is available at https://github.com/sysbio-bioinf/QuantumBooleanNetworks (https://doi.org/10.5281/zenodo.7560006). This includes transpilation and simulation seeds in all scripts for generating the resulting visualizations. The general structure of the circuits is also given in the supplemental information.

## Supplemental information

Document S1. Figures S1–S7 and Tables S1–S3

Document S2. Article plus supplemental information

## Footnotes

Supplemental information can be found online at https://doi.org/10.1016/j.patter.2023.100705.
