# Supplementary material for: Leveraging quantum computing for dynamic analyses of logical networks in systems biology
Source: Patterns (N Y). 2023 Mar 10;4(3):100705. doi: 10.1016/j.patter.2023.100705 (PMC10028428; doi:10.1016/j.patter.2023.100705)
Supplement: Document S1. Figures S1–S7 and Tables S1–S3 [file mmc1.pdf]

Patterns, Volume *4*

Supplemental information

Leveraging quantum computing for dynamic analyses of logical networks in systems biology

Felix M. Weidner, Julian D. Schwab, Sabine Wölk, Felix Rupprecht, Nensi Ikonomi, Silke D. Werle, Steve Hoffmann, Michael Kühl, and Hans A. Kestler

1 Relevant quantum gates for QBN circuits

The following section shows the matrix representations of the most relevant quantum gates for the implementation of a QBN in the computational basis $|0\rangle = \begin{bmatrix} 1 \\ 0 \end{bmatrix}, |1\rangle = \begin{bmatrix} 0 \\ 1 \end{bmatrix}$. Note that the little-endian ordering of qubits used by Qiskit differs from the order frequently used in the literature. In Qiskit, states are ordered as $|q_{n-1}, ..., q_1, q_0\rangle$, and all the following matrices follow this convention. The operator corresponding to a classical `NOT` ($\neg$) can be implemented using a single-qubit X gate, which operates on a qubit state vector as given by the matrix

$$\mathrm{X}\,|q_0\rangle = \neg\,|q_0\rangle = \begin{bmatrix} 0 & 1 \\ 1 & 0 \end{bmatrix} |q_0\rangle \tag{1}$$

The implementation of `AND` ($\wedge$) and `OR` ($\vee$) operations is achieved using the Toffoli gate[1], also known as the CCX or controlled-controlled-NOT gate. This gate operates on three qubits q_0, q_1, q_2, flipping the target q_2 if and only if both control qubits are in the state $|1\rangle$.

$$\begin{aligned}
\mathrm{CCX}\,|q_2, q_1, q_0\rangle &= |(q_0 \wedge q_1) \oplus q_2, q_1, q_0\rangle && \text{General case} && (2)\\
\mathrm{CCX}\,|0, q_1, q_0\rangle &= |q_0 \wedge q_1, q_1, q_0\rangle && \text{AND} && (3)\\
\mathrm{CCX}\,|0, \neg q_1, \neg q_0,\rangle &= |\neg(q_0 \vee q_1), \neg q_1, \neg q_0\rangle && \text{OR} && (4)\\
\mathrm{CCX}\,|q_2, 1, 1\rangle &= |\neg q_2, 1, 1\rangle && \text{NOT} && (5)
\end{aligned}$$

where $\oplus$ indicates addition modulo 2. Its matrix representation is

$$\mathrm{CCX} = \begin{bmatrix} 1 & 0 & 0 & 0 & 0 & 0 & 0 & 0 \\ 0 & 1 & 0 & 0 & 0 & 0 & 0 & 0 \\ 0 & 0 & 1 & 0 & 0 & 0 & 0 & 0 \\ 0 & 0 & 0 & 0 & 0 & 0 & 0 & 1 \\ 0 & 0 & 0 & 0 & 1 & 0 & 0 & 0 \\ 0 & 0 & 0 & 0 & 0 & 1 & 0 & 0 \\ 0 & 0 & 0 & 0 & 0 & 0 & 1 & 0 \\ 0 & 0 & 0 & 1 & 0 & 0 & 0 & 0 \end{bmatrix}. \tag{6}$$

The Toffoli gate alone is thus sufficient for implementing Boolean logic. However, negations will still be implemented using the one-qubit X gate. Generally, the Toffoli gate flips its target qubit q_2 if both its control qubits are in the $|1\rangle$ state. A generalisation of this gate with multiple control qubits is referred to as the multi-controlled-NOT or MCX gate.

The need for implementing these operations using a third qubit arises from the requirement of quantum operators to be unitary, which also implies reversibility. This property of unitarity is a consequence of gate operations needing to preserve the normalisation of the quantum state[2]. Additional relevant quantum gates are the Hadamard or H-gate, which transforms the $|0\rangle$ state into an equally weighted superposition of $|0\rangle$ and $|1\rangle$, and the $R_y(\theta)$ gate, which rotates the qubit states around the y-axis by an angle of θ (for the analyses performed in this work, the $R_x(\theta)$ gate would serve the same purpose). Lastly, the SWAP gate swaps the state of two qubits.

$$H = \frac{1}{\sqrt{2}} \begin{bmatrix} 1 & 1 \\ 1 & -1 \end{bmatrix} \tag{7}$$

$$R_y(\theta) = \begin{bmatrix} \cos(\theta/2) & -\sin(\theta/2) \\ \sin(\theta/2) & \cos(\theta/2) \end{bmatrix} \tag{8}$$

$$SWAP = \begin{bmatrix} 1 & 0 & 0 & 0 \\ 0 & 0 & 1 & 0 \\ 0 & 1 & 0 & 0 \\ 0 & 0 & 0 & 1 \end{bmatrix} \tag{9}$$

The quantum counting circuit shown in Supplementary Figure 5 below requires the use of controlled gates. In general, a unitary operator $\hat{U}$ can be turned into a controlled $c\hat{U}$ operation as

$$c\hat{U} = \begin{bmatrix} 1 & 0 & 0 & 0 \\ 0 & u_{00} & 0 & u_{01} \\ 0 & 0 & 1 & 0 \\ 0 & u_{10} & 0 & u_{11} \end{bmatrix} \tag{10}$$

2 Synchronous and asynchronous update circuit

The following Supplementary Figure 1 shows quantum circuits for synchronous and asynchronous updates of a toy model Boolean network. The rules of this model are

$$x_0(t+1) = \neg x_1(t) \vee x_2(t) \tag{11}$$
$$x_1(t+1) = x_0(t) \wedge x_1(t) \wedge x_2(t) \tag{12}$$
$$x_2(t+1) = x_2(t). \tag{13}$$

Both updating schemes perform the same number of operations, but their order differs in general due to the randomization in the asynchronous update scheme. Given asynchronous updates, qubits in the output register can also be used as inputs for the remaining Boolean functions.

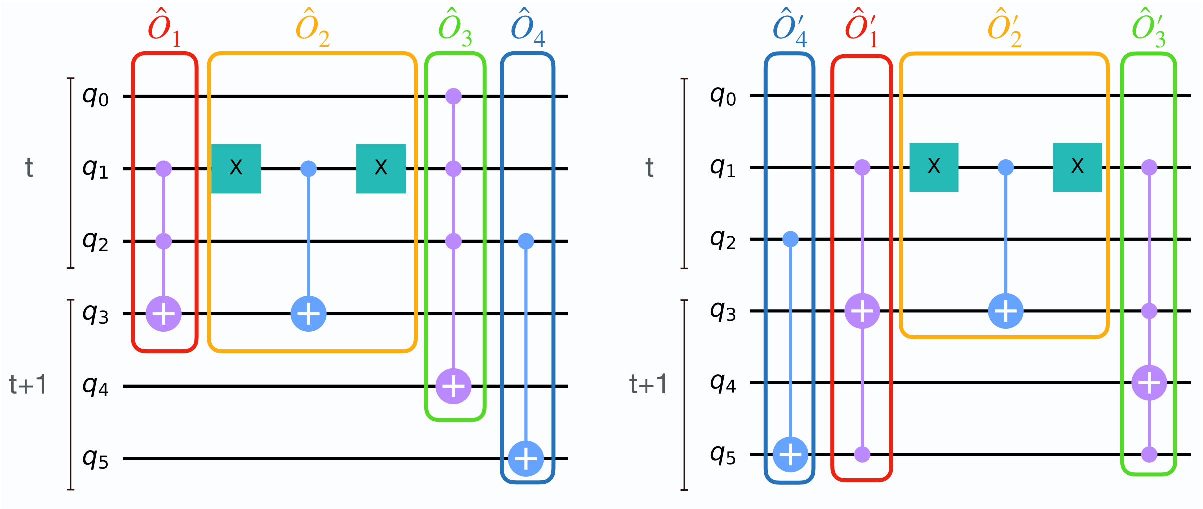

Supplementary Figure 1: **Quantum circuits for a single state transition using synchronous and asynchronous updating.** Synchronous (left) and asynchronous (right) update circuits for a three gene toy model. The asynchronous circuits applies the same gate operations $\hat{O}_1$ to $\hat{O}_4$ as the synchronous one, however their order is shifted. The asynchronous update order in this case is $x_2 \to x_0 \to x_1$. The output qubits q_3 to q_5 are also used as inputs for the remaining updates.

3 Quantum circuits for performing multiple state transitions

Quantum circuits for performing multiple state transitions were implemented using a scheme that matches a time step to a particular register. For this purpose, the input register of n qubits is initialized with a layer of Hadamard gates to create a uniform superposition state. This is followed by the logic gates implementing the Boolean rules, with their outputs being on a separate ancilla register which was initialized in the $|0\rangle$ state. This procedure is repeated, yielding a circuit with $(T+1)n$ qubits for T transitions in an n component network. The final register is then measured. Biological systems are scale-free and exist in the critical phase[3,4]. Such networks show a transient time to attractors that scales linearly with network size[5]. Thus, the circuit width for repeated state transitions scales as $\mathcal{O}(n^2)$.
A second scheme was used for generating the results for the $n = 10$ cell cycle network[6], shown below in Figure 3. This was done due to the high number of qubits required for simulating the statevector of this network for multiple transitions.
In this approach, a $2n$-qubit circuit is reinitialized with a state $|\Psi\rangle$ which is determined by the probability distribution obtained from repeated measurements of the previous transition. This reinitialization is costly to implement and leads to less accurate results for increasing values of T. Therefore, it is recommended to use the single circuit scheme with multiple registers for use on real QPUs.

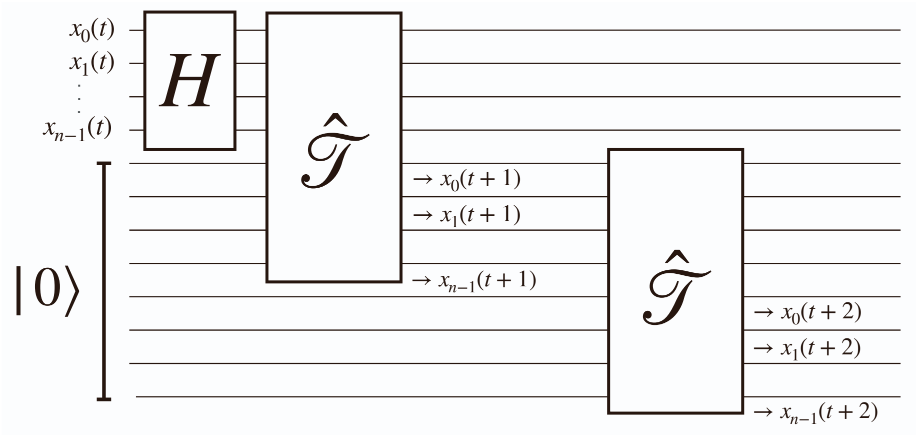

Supplementary Figure 2: **Quantum circuit for performing repeated state transitions across multiple registers.** Scheme for performing T quantum state transitions in a single circuit of $(T+1)n$ qubits in a QBN of n compounds, starting from a uniform superposition state.

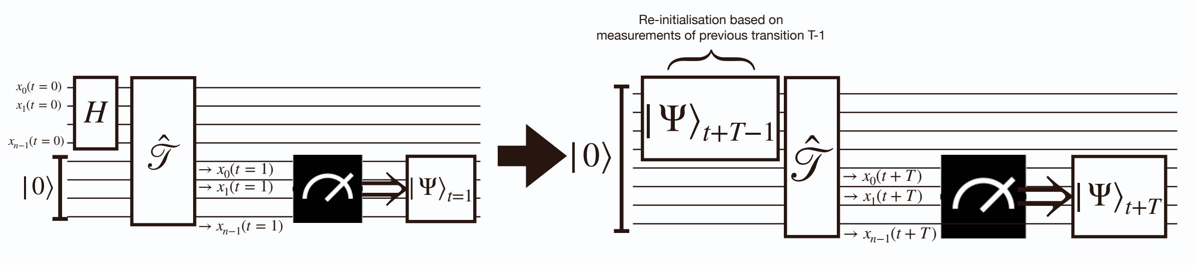

Supplementary Figure 3: **Quantum circuit for peforming a single state transition using re-initialization.** Scheme for simulating multiple quantum state transition using $2n$ qubits. The first transition is performed starting from a uniform superposition states. For further transitions, the obtained network state $|\Psi(t+1)\rangle$ is measured repeatedly, and the obtained weights are used to reinitialise the circuit corresponding to the obtained probability distribution. This procedure only measures the absolute values of the weights but not their phases. This reinitialisation scheme was used on a statevector simulator in order to show results from the $n = 10$ network of Fauré et al.[6]. For implementations on a real QPU, the implementation shown in Supplementary Figure 2 should be used.

4 Analysis of the cell cycle network of Fauré et al.

In addition to the mammalian cortical area development network of Giacomantonio and Goodhill[7], we further analysed quantum circuits for conducting state transitions in the $n = 10$ cell cycle network of Fauré et al.[6].

Having performed a single quantum state transition, $89/1024 \approx 8.7\%$ of states remain as possible measurement results. After a total of $T = 9$ transitions, only 8 states remain. These correspond to the attractor states present in the classical model, which consist of a single state attractor whose basin makes up 50% of the STG as well as a 7-step cyclic attractor. The probabilities of measuring one of these 7 states add up to the remaining 50%. In a QBN simulation with 10000 measurements, probabilities of $45.92 \pm 0.5\%$ for the single state and a cumulative $54.08 \pm 0.5\%$ for the for the cyclic attractor were obtained. Given the qubit requirement for simulating $T = 9$ transitions, this simulation for the cell cyle network was performed using the scheme shown in Supplementary Figure 3, rather than that shown in Supplementary Figure 2 which was used for the mammalian cortical area development network.

We also performed simulations varying the initial activity of all 10 components, analogous to the inset of Figure 2b shown in the manuscript.

The following Supplementary Figure 4 compares the results obtained in these networks. This shows that in the cell cycle network, only the initial bias of the component CycD is relevant for the obtained attractor probability (the unbiased values are indicated by dashed black lines). In contrast, in the mammalian cortical area development network, the bias of multiple components can have an influence on the attractor probability, however none has an impact that is as large as the bias of the component CycD in the cell cycle network of Fauré et al.[6].

In addition to the simulation of quantum circuits, an equivalent classical simulation has been performed using the R package BoolNet[8]. Here, for simulating the influence of initial expression biases, 10000 starting states were generated where the probability of being expressed was 50% for all but the biased component.

The probability $p_{on}(\theta)$ of the biased component being expressed in a given starting state can be calculated from the angle θ as

$$p_{on}(\theta) = \sin^2(\theta/2) \tag{14}$$

corresponding to an application of the $R_y(\theta)$ matrix as given in section 1, keeping in mind that probabilities are proportional to the square of a state's amplitude.
In the same manner, one can also assign biases to multiple components at once while leaving others in an unbiased starting state, depending on available prior knowledge.

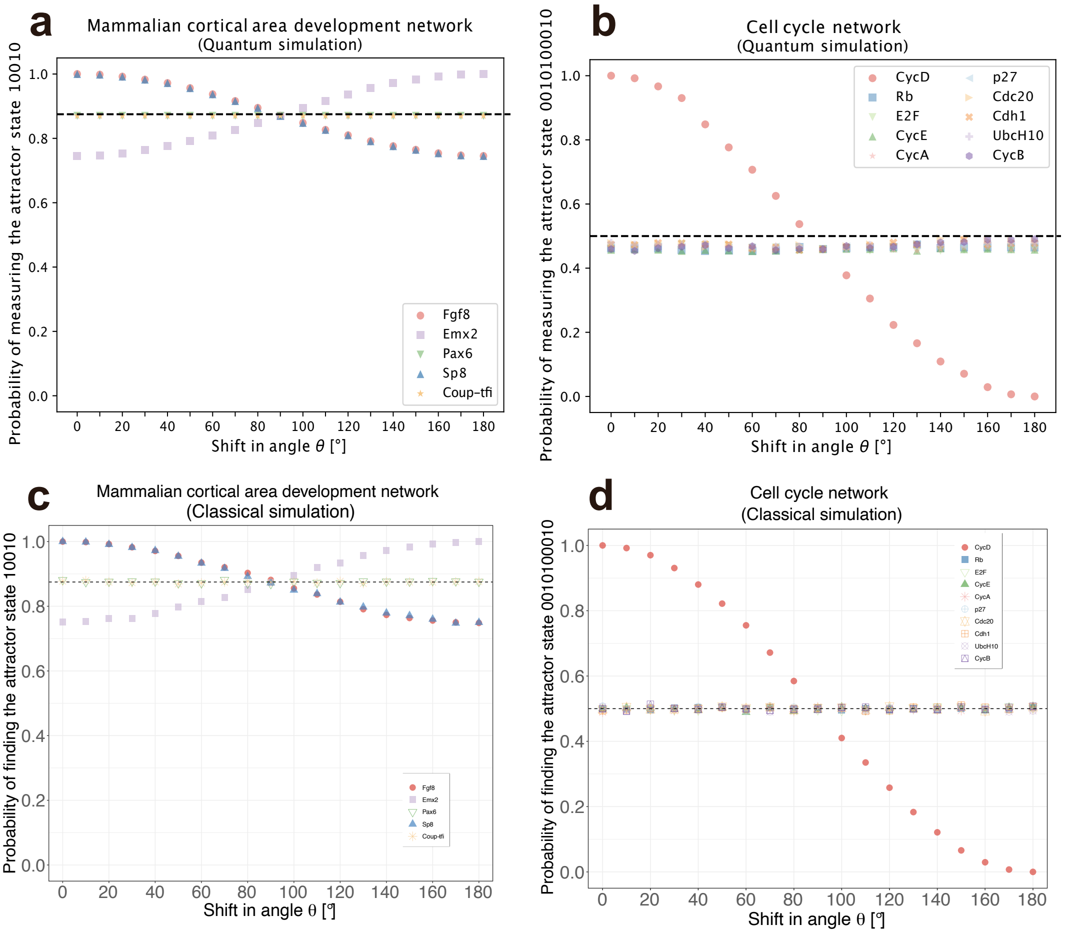

Supplementary Figure 4: **Shift in basin size with continuous variation of input activity**. Change in the basin size of single state attractors in the mammalian cortical area development network[7] (**a**, **c**) and the cell cycle network[6] (**b**, **d**). Subfigures **a** and **b** show simulations of quantum circuits where initial biases were induced by $R_y(\theta)$ gates for every component in the network. The same analysis was performed on the classical networks as shown in subfigures **c** and **d**. Here, instead of 10000 measurements of a quantum circuit, an equivalent number of starting states was generated, with the probability of any component being expressed at 50%. The only exception is the single biased components, which had it's probability of expression calculated to correspond to the chosen angle θ. In a classical exhaustive and unbiased simulation, the basins of these attractors make up 87.5% (a) and 50% (b) of their respective network's STGs, indicated by dashed black lines.

5 Attractor distribution for double perturbation using superposition states

The use of R_y gates can be further extended to include perturbations with superposition states. That is, a given component will not be updated according to its Boolean rule, but will retain a previously specified superposition state. Thus it becomes possible to simulate overexpressions and knockouts of different strengths, in which a component will always retain a non-zero probability of being active or inactive.

To demonstrate this, we have performed superposition perturbations on the mammalian cortical area development network[7]. We biased the component Pax6 towards overexpression with an $R_y(\theta = 3\pi/4)$ perturbation and simultaneously biased the component Coup-tfi towards a knockout with $R_y(\theta = \pi/4)$. These components were chosen as an example, given that they yield an attractor state that is not present in the unperturbed system.

Classically, a double perturbation of these components reduces the number of possible transitions in the STG by 2^2, and yields the two single state attractors 00100 and 01101. The basin sizes of these attractors are 87.5% (7/8) and 12.5% (1/8) respectively. The superposition perturbations likewise find these attractors. However, there also appear additional attractors with small but non-zero probabilities, which correspond to states which appear in the complementary Pax6-KO + Coup-tfi-OE perturbation, or from double OE and double KO perturbations. That is, superposition perturbations are equivalent to performing simultaneous overexpression and knockout simulations of a component. This section lists all obtained attractors in order of decreasing probability of measurement, as well as the occurrences of these attractors in classical perturbation simulations.

Matching the bias of the superposition perturbations, the Pax6-OE + Coup-tfi-KO attractor has the highest probability while its opposite, Pax6-KO + Coup-tfi-OE, achieves the lowest weight. Thus, one analysis in a QBN with $P = 2$ perturbed components has yielded a union set of attractors from all 2^P classical multi-component perturbation simulations.

Supplementary Table 2 shows the distribution of attractors given a double perturbation using superposition states of the nodes Pax6 and Coup-tfi in the mammalian cortical area development network[7]. The first node was biased towards overexpression using an $R_y(\theta = 3\pi/4)$ gate and the second towards a knockout with an $R_y(\theta = \pi/4)$ perturbation.

Supplementary Table 1 displays the attractors and basin sizes obtained in all four double perturbations of these two nodes in a classical Boolean network.

**Perturbations**		**Attractors (Basin sizes)**	
Pax6-OE	Coup-tfi-KO	00100 (87.5%)	01101 (12.5%)
Pax6-OE	Coup-tfi-OE	10100 (87.5%)	11101 (12.5%)
Pax6-KO	Coup-tfi-KO	00000 (87.5%)	01001 (12.5%)
Pax6-KO	Coup-tfi-OE	10010 (87.5%)	11001 (12.5%)

Supplementary Table 1: **Basin distribution for all four possible two-node perturbations in a classical Boolean network.** Attractor distributions for various double perturbations in the mammalian cortical area development network[7].

**Attractor**	**00100**	**00000**	**10100**	**01101**	**10010**	**11101**	**01001**	**11001**
**Probability**	$63.4 \pm 0.5\%$	$11.2 \pm 0.3\%$	$10.9 \pm 0.3\%$	$9.3 \pm 0.3\%$	$1.8 \pm 0.1\%$	$1.8 \pm 0.1\%$	$1.5 \pm 0.1\%$	$0.2 \pm 0.05\%$
**Occurrence**	Pax6-OE Coup-tfi-KO	Pax6-KO Coup-tfi-KO	Pax6-OE Coup-tfi-OE	Pax6-OE Coup-tfi-KO	Pax6-KO Coup-tfi-OE	Pax6-OE Coup-tfi-OE	Pax6-KO Coup-tfi-KO	Pax6-KO Coup-tfi-OE

Supplementary Table 2: **Basin distribution for a two-node perturbation using superposition states.** Attractor distribution of a double superposition perturbation in the mammalian cortical area development network[7]. The measured states include all attractors which classically appear in all possible overexpression/knockout combinations of these two perturbed components.

6 Circuit for Grover amplitude amplification of predecessor states

Supplementary Figure 5 shows a quantum circuit implementing a Grover search for predecessor states of a given marked state (such as an attractor). The circuit consists of an n qubit input register which is initialized with a uniform superposition state using Hadamard gates. Then, T state transitions are performed, yielding their output on another n qubit register. For the phase flip of the marked state ω induced by the oracle $\hat{U}_\omega$, the marked state is transformed to the $|11...1\rangle$ state via a layer of X gates. This is done by applying an X gate on a qubit if the corresponding entry in the marked state is 0. Thus, the MCX gate will flip the state of a separate qubit prepared in the $|-\rangle = \mathrm{HX}\,|0\rangle = \frac{1}{\sqrt{2}}(|0\rangle - |1\rangle)$ state only for the marked state.
That is, the general black box oracle used in Grover's algorithm is here implemented by Boolean dynamics and the marking of an attractor, carrying the resulting phase difference back to the initial register via uncomputing.
The diffuser $\hat{D}$ then modifies the original input register, which is measured to yield a probability distribution in which the predecessor states' weights have been amplified.

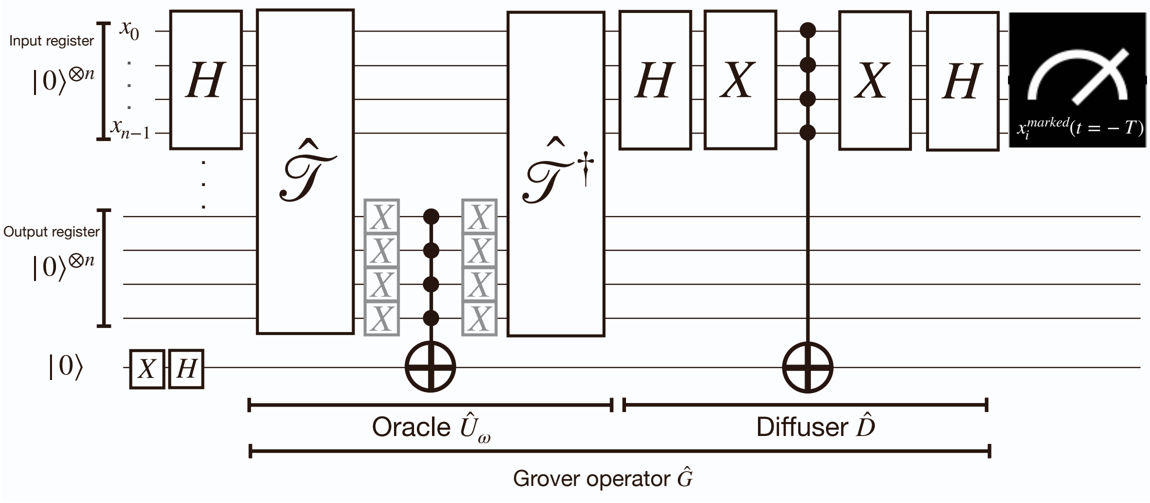

Supplementary Figure 5: **Circuit for amplifying the probability of measuring predecessors of a marked state in the STG using a Grover search.** A single iteration of Grover's algorithm is applied to the search for predecessor states. An initial uniform superposition state is prepared, followed by an oracle which includes the state transition circuit. A separate qubit in the $|-\rangle$ state is used to kick back the phase flip induced by the oracle towards the initial register. The layers of grey X gates depend on the marked state $|\omega\rangle$. An X gate is only applied if the corresponding value of the marked state for this network component is 0. Thus, these gates perform the transformation $|\omega\rangle \to |111...1\rangle$, activating the MCX gate.

7 Circuit for quantum counting of predecessor states

The Grover operator $\hat{G}$ shown in Supplementary Figure 5 is used as a component for a quantum counting algorithm as presented by Brassard et al.[9]. Here, the Grover operator is turned into a controlled operation, performing an increasing number of iterations controlled by a set of r qubits in a readout register. Lastly, an inverted quantum Fourier transformation ($QFT^{\dagger}$) is applied to this register.
The integer representation i of the measured bitstring b of length r can be converted into an estimate for a phase angle ϕ and then into the number of non-predecessors $N - M$ as specified in equation (16).

$$\phi = \frac{i \cdot 2 \cdot \pi}{2^r} \tag{15}$$

$$N - M = N \cdot \sin^2(\phi/2) \tag{16}$$

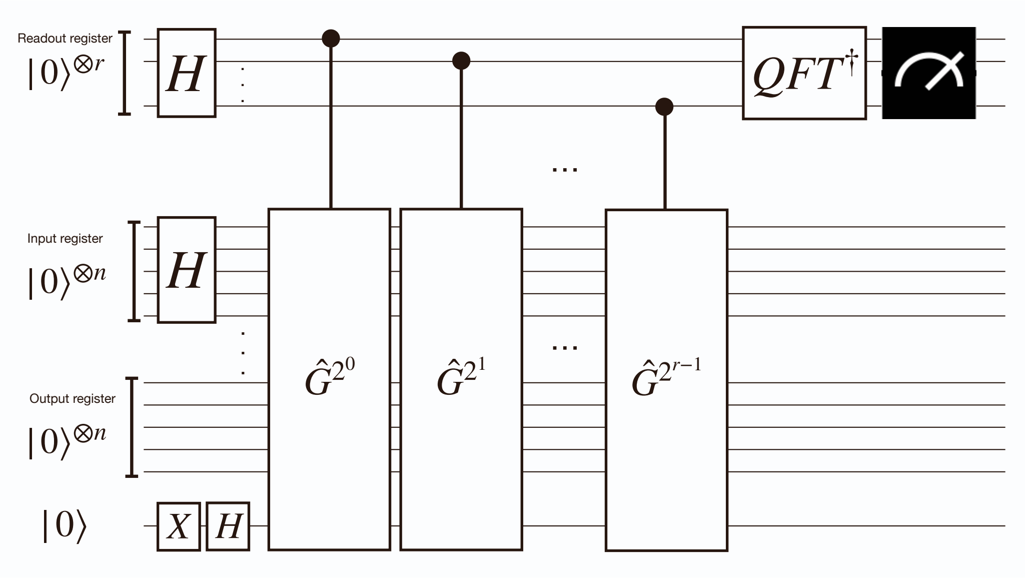

Supplementary Figure 6: **Circuit for counting the number of predecessors of a marked state in the STG.** Circuit for the implementation of a quantum counting algorithm. The repeated Grover operators $\hat{G}^{2^i}$ are constructed as in Figure 5. An inverted Quantum Fourier Transformation ($QFT^{\dagger}$) is performed before measuring the output of an r qubit readout register, yielding an estimate for the number of non-predecessor states $N - M$.

8 Fraction M/N of predecessor states across networks

The advantage of quantum algorithms in the search for a particular set of states in a large state space is particularly relevant when this ratio M/N of solutions to state space size is small. Thus, this ratio was investigated across a set of biologically motivated Boolean network models.
The selected networks were extracted from *https://cellcollective.org/* [10] as well as from pubMed by using the search term "Boolean network model" (status 24.05.2017) and are listed in Supplementary Table 3.
For the calculation of the reduction speed in possible measurement states after some number of transitions, we analysed the transition tables of the networks using the BoolNet R-package[8]. Since exhaustive attractor searches are limited to at most $n = 29$ components in this package, only networks which did not exceed this limit were analysed.
We further excluded networks for which analyses of the full transition table could not be conducted in under 24 h of computation time.
Networks were also not considered in case that their dynamics could be reduced to a set of input components. Lastly, the PoweRlaw R-package[11] was used to check for scale-free degree distributions, retaining networks with p-values above a threshold of p $= 0.1$ as described by Clauset et al.[12].
In total, this set of networks has an average of 15.5 ± 5.4 components, with an average of 38.6 ± 17.1 interactions.
Supplementary Figure 7 shows the fraction of predecessor states M relative to the size of the state space $N = 2^n$ of a network for a given number of inverted state transitions. In a given network, the specified number of inverted transitions was performed starting from all attractor states (whether fix points or part of cyclic attractors), averaging the results. This was performed across all networks.

We note that while a single inverted state transition yielded a median value of $M/N = 0.003$, this median approached a value of $M/N = 0.1$ as the number of inverted transitions was increased. This occurs due to the small size of the analyzed networks, indicating that the inverted transitions have arrived at most of the Garden of Eden states.
It is possible however that larger networks may yield smaller M/N ratios, as after $T^{inv} = 1$, the Pearson correlation of the obtained fractions of predecessor states with network size was -0.714.

Authors and Year	Number of components	Number of interactions
Azpeitia et al., 2013 [13]	14	24
Brandon et al., 2015 [14]	22	40
Dahlhaus et al., 2016 [15]	23	47
Davila-Velderrain et al., 2015 [16]	13	42
Fauré et al., 2006 [6]	10	35
García-Gómez et al., 2017 [17]	16	39
Giacomantonio and Goodhill, 2010 [7]	5	14
Gupta et al., 2007 [18]	16	22
Herrmann et al., 2012 [19]	15	38
Irons, 2009 [20]	18	59
Krumsiek et al., 2011 [21]	11	30
MacLean and Studholme, 2010 [22]	7	12
Marques-Pita and Rocha, 2013 [23]	17	32
Martinez-Sanchez et al., 2015 [24]	12	72
Méndez and Mendoza, 2016 [25]	22	44
Méndez-López et al., 2017 [26]	9	34
Mendoza and Xenarios, 2006 [27]	23	38
Orlando et al., 2008 [28]	9	19
Ortiz-Gutiérrez et al., 2015 [29]	14	66
Ríos et al., 2015 [30]	19	79
Saadatpour et al., 2011 [31]	18	43
Sahin et al., 2009 [32]	20	52
Sankar et al., 2011 [33]	20	30
Siegle et al., 2018 [34]	23	55
Sridharan et al., 2012 [35]	19	31
Sun et al., 2014 [36]	8	13
Todd and Helikar, 2012 [37]	20	46
Yousefi and Dougherty, 2013 [38]	10	24

Supplementary Table 3: **List of analysed network models.** Authors of the 28 selected networks, including the number of components and interactions in each model.

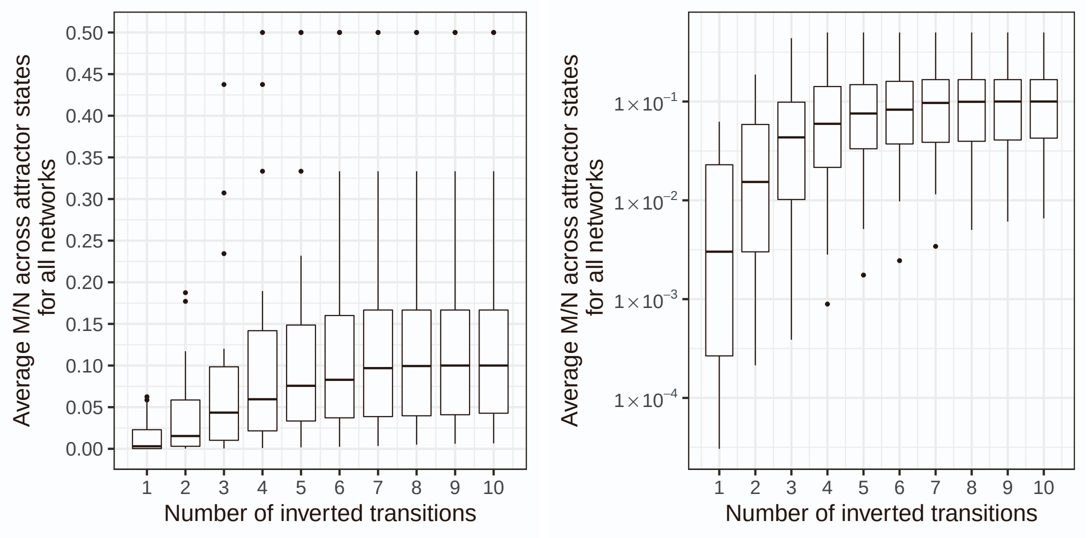

(a) linear scale (b) logarithmic scale

Supplementary Figure 7: **Fraction of predecessor states across all attractor states of all analyzed networks.** Box plots showing the fraction of predecessor states relative to the size of the state space M/N for an increasing number of inverted state transitions. The obtained ratio of predecessors was averaged across all attractor states of a network. Results are shown for all 28 analyzed networks on both a linear and logarithmic scale.

References

[1] Fredkin, E. and Toffoli, T. (1982). Conservative Logic. Int. J. Theor. Phys., 21(3), pp. 219–253. doi:10.1007/BF01857727.

[2] Hey, T. (1999). Quantum Computing: An Introduction. Comput. & Control Eng. J., 10(3), pp. 105–112. doi:10.1049/ccej:19990303.

[3] Albert, R. (2005). Scale-free networks in cell biology. J. Cell Sci., 118(21), pp. 4947–4957. doi:10.1242/jcs.02714.

[4] Kauffman, S.A. and Johnsen, S. (1991). Coevolution to the Edge of Chaos: Coupled Fitness Landscapes, Poised States, and Coevolutionary Avalanches. J. Theor. Biol., 149(4), pp. 467–505. doi:10.1016/S0022-5193(05)80094-3.

[5] Aldana, M. (2003). Boolean dynamics of networks with scale-free topology. Phys. D Nonlinear Phenom., 185(1), pp. 45–66. doi:10.1016/S0167-2789(03)00174-X.

[6] Fauré, A., Naldi, A., Chaouiya, C., and Thieffry, D. (2006). Dynamical analysis of a generic Boolean model for the control of the mammalian cell cycle. Bioinformatics, 22(14), pp. e124–e131. doi:10.1093/bioinformatics/btl210.

[7] Giacomantonio, C.E. and Goodhill, G.J. (2010). A Boolean Model of the Gene Regulatory Network Underlying Mammalian Cortical Area Development. PLoS Comput. Biol., 6(9), p. e1000936. doi:10.1371/journal.pcbi.1000936.

[8] Müssel, C., Hopfensitz, M., and Kestler, H.A. (2010). BoolNet—an R package for generation, reconstruction and analysis of Boolean networks. Bioinformatics, 26(10), pp. 1378–1380. doi:10.1093/bioinformatics/btq124.

[9] Brassard, G., Høyer, P., and Tapp, A. (1998). Quantum counting. In Lect. Notes Comput. Sci. (Springer), (pp. 820–831). doi:10.1007/BFb0055105.

[10] Helikar, T., Kowal, B., McClenathan, S., Bruckner, M., Rowley, T., Madrahimov, A., Wicks, B., Shrestha, M., Limbu, K., and Rogers, J.A. (2012). The Cell Collective: Toward an open and collaborative approach to systems biology. BMC Syst. Biol., 6(1), p. 96. doi:10.1186/1752-0509-6-96.

[11] Gillespie, C. (2015). Fitting Heavy Tailed Distributions: The poweRlaw Package. J. Stat. Softw., 64, pp. 1–16. doi:10.18637/jss.v064.i02.

[12] Clauset, A., Shalizi, C.R., and Newman, M.E. (2009). Power-Law Distributions in Empirical Data. SIAM Rev., 51(4), pp. 661–703. doi:10.1137/070710111.

[13] Azpeitia, E., Weinstein, N., Benítez, M., Mendoza, L., and Alvarez-Buylla, E.R. (2013). Finding missing interactions of the Arabidopsis thaliana root stem cell niche gene regulatory network. Front. Plant Sci., 4, p. 110. doi:10.3389/fpls.2013.00110.

[14] Brandon, M., Howard, B., Lawrence, C., and Laubenbacher, R. (2015). Iron acquisition and oxidative stress response in aspergillus fumigatus. BMC Syst. Biol., 9(1), p. 19. doi:10.1186/s12918-015-0163-1.

[15] Dahlhaus, M., Burkovski, A., Hertwig, F., Mussel, C., Volland, R., Fischer, M., Debatin, K.M., Kestler, H.A., and Beltinger, C. (2016). Boolean modeling identifies Greatwall/MASTL as an important regulator in the AURKA network of neuroblastoma. Cancer Lett., 371(1), pp. 79–89. doi:10.1016/j.canlet.2015.11.025.

[16] Davila-Velderrain, J., Villarreal, C., and Alvarez-Buylla, E.R. (2015). Reshaping the epigenetic landscape during early flower development: induction of attractor transitions by relative differences in gene decay rates. BMC Syst. Biol., 9(1), p. 20. doi:10.1186/s12918-015-0166-y.

[17] García-Gómez, M.L., Azpeitia, E., and Álvarez-Buylla, E.R. (2017). A dynamic genetic-hormonal regulatory network model explains multiple cellular behaviors of the root apical meristem of Arabidopsis thaliana. PLoS Comput. Biol., 13(4), p. e1005488. doi:10.1371/journal.pcbi.1005488.

[18] Gupta, S., Bisht, S.S., Kukreti, R., Jain, S., and Brahmachari, S.K. (2007). Boolean network analysis of a neurotransmitter signaling pathway. J. Theor. Biol., 244(3), pp. 463–469. doi:10.1016/j.jtbi.2006.08.014.

[19] Herrmann, F., Groß, A., Zhou, D., Kestler, H.A., and Kühl, M. (2012). A Boolean Model of the Cardiac Gene Regulatory Network Determining First and Second Heart Field Identity. PLoS One, 7(10), p. e46798. doi:10.1371/journal.pone.0046798.

[20] Irons, D. (2009). Logical analysis of the budding yeast cell cycle. J. Theor. Biol., 257(4), pp. 543–559. doi:10.1016/j.jtbi.2008.12.028.

[21] Krumsiek, J., Marr, C., Schroeder, T., and Theis, F.J. (2011). Hierarchical Differentiation of Myeloid Progenitors Is Encoded in the Transcription Factor Network. PLoS One, 6(8). doi:10.1371/journal.pone.0022649.

[22] MacLean, D. and Studholme, D.J. (2010). A Boolean Model of the Pseudomonas syringae hrp Regulon Predicts a Tightly Regulated System. PLoS One, 5(2). doi:10.1371/journal.pone.0009101.

[23] Marques-Pita, M. and Rocha, L.M. (2013). Canalization and Control in Automata Networks: Body Segmentation in Drosophila melanogaster. PLoS One, 8(3). doi:10.1371/journal.pone.0055946.

[24] Martinez-Sanchez, M.E., Mendoza, L., Villarreal, C., and Alvarez-Buylla, E.R. (2015). A Minimal Regulatory Network of Extrinsic and Intrinsic Factors Recovers Observed Patterns of CD4+ T Cell Differentiation and Plasticity. PLOS Computational Biology, 11(6). doi:10.1371/journal.pcbi.1004324.

[25] Méndez, A. and Mendoza, L. (2016). A Network Model to Describe the Terminal Differentiation of B Cells. PLoS Comput. Biol., 12(1). doi:10.1371/journal.pcbi.1004696.

[26] Méndez-López, L.F., Davila-Velderrain, J., Domínguez-Hüttinger, E., Enríquez-Olguín, C., Martínez-García, J.C., and Alvarez-Buylla, E.R. (2017). Gene regulatory network underlying the immortalization of epithelial cells. BMC Syst. Biol., 11(1), p. 24. doi:10.1186/s12918-017-0393-5.

[27] Mendoza, L. and Xenarios, I. (2006). A method for the generation of standardized qualitative dynamical systems of regulatory networks. Theor. Biol. Med. Model., 3(1), p. 13. doi:10.1186/1742-4682-3-13.

[28] Orlando, D.A., Lin, C.Y., Bernard, A., Wang, J.Y., Socolar, J.E., Iversen, E.S., Hartemink, A.J., and Haase, S.B. (2008). Global control of cell-cycle transcription by coupled CDK and network oscillators. Nature, 453(7197), pp. 944–947. doi:10.1038/nature06955.

[29] Ortiz-Gutiérrez, E., García-Cruz, K., Azpeitia, E., Castillo, A., de la Paz Sanchez, M., and Álvarez-Buylla, E.R. (2015). A Dynamic Gene Regulatory Network Model That Recovers the Cyclic Behavior of Arabidopsis thaliana Cell Cycle. PLoS Comput. Biol., 11(9). doi:10.1371/journal.pcbi.1004486.

[30] Ríos, O., Frias, S., Rodríguez, A., Kofman, S., Merchant, H., Torres, L., and Mendoza, L. (2015). A Boolean network model of human gonadal sex determination. Theor. Biol. Med. Model., 12(1), p. 26. doi:10.1186/s12976-015-0023-0.

[31] Saadatpour, A., Wang, R.S., Liao, A., Liu, X., Loughran, T.P., Albert, I., and Albert, R. (2011). Dynamical and Structural Analysis of a T Cell Survival Network Identifies Novel Candidate Therapeutic Targets for Large Granular Lymphocyte Leukemia. PLoS Comput. Biol., 7(11). doi:10.1371/journal.pcbi.1002267.

[32] Sahin, Ö., Fröhlich, H., Löbke, C., Korf, U., Burmester, S., Majety, M., Mattern, J., Schupp, I., Chaouiya, C., Thieffry, D., et al. (2009). Modeling ERBB receptor-regulated G1/S transition to find novel targets for de novo trastuzumab resistance. BMC Syst. Biol., 3(1), p. 1. doi:10.1186/1752-0509-3-1.

[33] Sankar, M., Osmont, K.S., Rolcik, J., Gujas, B., Tarkowska, D., Strnad, M., Xenarios, I., and Hardtke, C.S. (2011). A qualitative continuous model of cellular auxin and brassinosteroid signaling and their crosstalk. Bioinformatics, 27(10), pp. 1404–1412. doi:10.1093/bioinformatics/btr158.

[34] Siegle, L., Schwab, J.D., Kühlwein, S.D., Lausser, L., Tümpel, S., Pfister, A.S., Kühl, M., and Kestler, H.A. (2018). A Boolean network of the crosstalk between IGF and Wnt signaling in aging satellite cells. PLoS One, 13(3). doi:10.1371/journal.pone.0195126.

[35] Sridharan, S., Layek, R., Datta, A., and Venkatraj, J. (2012). Boolean modeling and fault diagnosis in oxidative stress response. BMC Genomics, 13(6), p. S4. doi:10.1186/1471-2164-13-S6-S4.

[36] Sun, M., Cheng, X., and Socolar, J.E. (2014). Regulatory logic and pattern formation in the early sea urchin embryo. J. Theor. Biol., 363, pp. 80–92. doi:10.1016/j.jtbi.2014.07.023.

[37] Todd, R.G. and Helikar, T. (2012). Ergodic Sets as Cell Phenotype of Budding Yeast Cell Cycle. PLoS One, 7(10). doi:10.1371/journal.pone.0045780.

[38] Yousefi, M.R. and Dougherty, E.R. (2013). Intervention in gene regulatory networks with maximal phenotype alteration. Bioinformatics, 29(14), pp. 1758–1767. doi:10.1093/bioinformatics/btt242.
